# Effects of probiotic supplements on cognition, anxiety, and physical activity in subjects with mild and moderate Alzheimer’s disease: A randomized, double-blind, and placebo-controlled study

**DOI:** 10.3389/fnagi.2022.1032494

**Published:** 2022-10-31

**Authors:** Camellia Akhgarjand, Zahra Vahabi, Sakineh Shab-Bidar, Farnaz Etesam, Kurosh Djafarian

**Affiliations:** ^1^Department of Clinical Nutrition, School of Nutritional Sciences and Dietetics, Tehran University of Medical Sciences, Tehran, Iran; ^2^Cognitive Neurology and Neuropsychiatry Division, Department of Psychiatry, Roozbeh Hospital, Tehran University of Medical Sciences, Tehran, Iran; ^3^Department of Geriatric, Ziaeeian Hospital, Tehran University of Medical Sciences, Tehran, Iran; ^4^Department of Community Nutrition, School of Nutritional Sciences and Dietetics, Tehran University of Medical Sciences, Tehran, Iran; ^5^Sina MS Research Center, Brain and Spinal Injury Research Center, Tehran University of Medical Sciences, Tehran, Iran

**Keywords:** Alzheimer’s disease, cognition, psychological test, probiotic, oral supplementation

## Abstract

Probiotics have been suggested as an effective adjuvant treatment for Alzheimer’s disease (AD) due to their modulating effect on the gut microbiota, which may affect the gut-brain axis. Therefore, we aimed to evaluate the effects of two different single-strain probiotics on cognition, physical activity, and anxiety in subjects with mild and moderate AD. Eligible patients (*n* = 90) with AD were randomly assigned to either of two interventions [*Lactobacillus rhamnosus* HA-114 (10^15^ CFU) or *Bifidobacterium longum* R0175 (10^15^ CFU)] or placebo group, receiving probiotic supplement twice daily for 12 weeks. The primary outcome of the study was cognitive function measured by using the two tests, namely, the Mini-Mental State Examination (MMSE) and the categorical verbal fluency test (CFT). Secondary outcomes included a performance in Activities of Daily Living (ADL), the Lawton Instrumental Activities of Daily Living (IADL) scale, and the Generalized Anxiety Disorder (GAD-7) scale. Linear mixed-effect models were used to investigate the independent effects of probiotics on clinical outcomes. After 12 weeks, MMSE significantly improved cognition (*P*_*Interaction*_ < 0.0001), with *post hoc* comparisons identifying significantly more improvement in the *B. longum* intervention group (differences: 4.86, 95% CI: 3.91–5.81; *P* < 0.0001) compared with both the placebo and *L. rhamnosus* intervention groups (differences: 4.06, 95% CI: 3.11–5.01; *P* < 0.0001). There was no significant difference between the two intervention groups (differences: −0.8, 95% CI: −1.74 to 0.14; *P* = 0.09). In conclusion, this trial demonstrated that 12-week probiotic supplementation compared with placebo had beneficial effects on the cognition status of patients with AD.

## Introduction

Alzheimer’s disease (AD) is a neurodegenerative and irreversible disease with a cognitive and behavioral impairment that accounts for 60–80% of dementia cases (Porsteinsson et al., 2021). Pathologically, the accumulation of beta-amyloid plaques outside neurons and hyperphosphorylated tau (pTau) tangles inside neurons are two of several brain changes associated with AD (Kincaid et al., 2021). Currently, more than 50 million individuals are living with dementia around the world, and as the population ages, this number will be tripled to 152 million by 2050 (International AsD, 2019); AD is the seventh leading cause of death among all diseases. In addition to cognitive impairment, AD has neuropsychiatric symptoms, of which the most common are anxiety and depression. Anxiety and depression can cause major problems for caregivers, reduce the patient’s quality of life, and increase hospitalization and mortality (Mendez, 2021). AD can also impair the patient’s ability to perform activities of daily living (ADL) and increase the patient’s dependence on caregivers (Clemmensen et al., 2020). The total annual cost of AD and other types of dementia in the United States is currently $305 billion and is expected to exceed $1.1 trillion by 2050 (Porsteinsson et al., 2021). The main risk factors for AD are age, genetics, and family history. However, studies have shown that obesity, type 2 diabetes, and gut dysbiosis are also risk factors for AD (Alzheimer’s Association, 2010). Unfortunately, the approved medicine is less effective on dementia, especially AD, and cannot stop the progression of the disease (Hazar et al., 2016); besides, these drugs have negative effects on the gut microbiome and can worsen the gut microbiome in the long-term. Therefore, adjuvant therapy with prebiotics/probiotics may prevent and/or cure gut dysbiosis and, therefore, can increase the therapeutic effects of AD drugs (Hort et al., 2020). Probiotics are a group of live microorganisms that are believed to have a variety of health benefits for the host such as anti-obesity, cancer-preventing effects, reducing anxiety, and increasing lifespan. They are usually added to yogurt or taken as a dietary supplement and are often called good or friendly bacteria. In addition, studies have shown that aging can decrease good bacteria in the gut especially *Bifidobacterium* and *Lactobacillus* (Oelschlaeger, 2010; Azm et al., 2017). Yet, few studies have been conducted on the relationship between probiotic supplements and cognitive status. Recent trials have used a single-strain probiotic to determine exactly which strain is more effective (Akbari et al., 2016; Azm et al., 2017; Musa et al., 2017; Leblhuber et al., 2018; Kobayashi et al., 2019). Based on the findings, this trial was designed to evaluate the effect of two different single-strain probiotics on cognitive status in subjects with mild and moderate AD.

## Materials and methods

### Study design

This was a 12-week multicenter, randomized, parallel, double-blind, and placebo-controlled clinical trial to observe the effect of probiotic supplementation on the cognitive status of patients with AD. This trial was performed in hospitals under the supervision of the Tehran University of Medical Sciences (TUMS), Iran. Our study was done according to the Declaration of Helsinki in 1975 and was also approved by the Ethics Committee of the Tehran University of Medical Sciences and registered on the Iranian Website for Registration of Clinical Trials IRCT (IRCT number: 20210513051277N1). We informed all the subjects about the risks and benefits of the study and reassured them that they could leave the study whenever they wanted. Written informed consent was received from all participants.

### Participants

A total of 90 older adults with mild and moderate AD took part in this clinical trial from October 2021 to March 2022. They were assigned to three groups (one group received *Lactobacillus rhamnosus* HA-114, the other one received *Bifidobacterium longum* R0175, and the last one took a placebo). The intervention started simultaneously in October 2021, and the last observation finished in May 2022. AD was diagnosed based on the NINDS-ADRDA criteria (Mckhann, 1984) and the received criteria from the National Institute on Aging Alzheimer’s Association (Jack et al., 2011). The inclusion criteria were as follows: aged 50–90 years, ability to tolerate oral medication, and mild or moderate AD based on the Functional Assessment Staging Tool (FAST) score 4-6B.

The exclusion criteria were as follows: (1) allergy to probiotic supplements, (2) reluctance to continue cooperation, (3) drastic changes in diet, (4) inflammatory diseases that lead to long-term use (more than 2 weeks) of anti-inflammatory drugs, (5) taking antibiotics, pre-probiotic or probiotic products, or multivitamin and mineral supplements during the intervention, (6) having any kind of infection or another disease like COVID-19, (7) changing the type of drug during the intervention, and (8) enrollment in any other intervention trial.

### Random assignment and masking

Stratified permute block randomization was performed in two categories based on age and sex by using the site www.randomization.com. In this method, one of the letters A, B, and C was assigned to each group and randomization was done in six blocks. Within each block, patients were randomly assigned to one of the three study groups in a 1:1:1 ratio. Probiotic supplements were coded by Lallemand Company and all the investigators, research coordinators, participants, caregivers, and data analysts were kept blind to treatment allocation throughout the trial.

### Intervention

Eligible participants were allocated in a 1:1 ratio to receive either of two interventions [a probiotic capsule containing *L. rhamnosus* HA (each capsule contained 10^15^ CFU probiotic) or a probiotic capsule containing *B. longum* R0175 (each capsule contained 10^15^ CFU probiotic) two times a day] or the placebo (one capsule containing xylitol, maltodextrin, and malic acid two times a day). All supplements provided by Lallemand Company in Canada were similar in color, taste, smell, and size. Caregivers were guided to store the supplements in the refrigerator and gave two capsules daily to patients, one after lunch and one after dinner. The supplements were delivered to caregivers monthly. To remind the regular consumption of supplements, a message was sent to caregivers daily. Caregivers were asked to return the medicine box and consumption of less than 80% of the supplement at the end of each month was considered non-compliant.

### Outcomes

The primary outcome was an assessment of cognitive function by using the two tests, namely, the Mini-Mental State Examination (MMSE) for illiterate patients and the categorical verbal fluency test (CFT), before and after the 12-week probiotic supplementation. Both tests were validated for the Iranian population (Seyedian et al., 2008; Clark et al., 2020).

Mini-Mental State Examination is the most widely used brief cognitive tool for evaluating a variety of cognitive disorders (Shulman et al., 2006) and it was published by Folstein et al. (1975). It is commonly used in clinical practice to assist clinicians in the diagnosis of dementia and delirium (Diniz et al., 2007). It has got 11 domains (orientation to time and place, registration of three words, calculation, language, and ability to draw a complex polygon), with a score range from 0 to 30, with higher numbers indicating better cognitive performance.

The CFT is used for assessing semantic and executive aspects of cognition (Lezak et al., 2004). It is a quick test that can be used to assess people with aphasia (Seniów et al., 2009) and neurodegenerative problems (Levy and Chelune, 2007; Arnott et al., 2010; Lepow et al., 2010). Previous research has found that dementia of AD affects CFT performance (Monsch et al., 1992; Henry et al., 2004). It usually includes two tasks, namely, category fluency (also known as semantic fluency) (Benton, 1968) and letter fluency (sometimes called phonemic fluency) (Newcombe, 1969). In the category fluency, patients are asked to say as many animal and fruit names as they can in 1 min. In the letter fluency, patients are also given 1 min to name the words beginning with the letter F. Based on the study of Shirdel et al. (2022), the letter F in English is equivalent to the letter Pe in Persian in terms of the number of words, that is why we used the letter Pe in letter fluency. The number of unique correct words was the patient’s score in each task (Benton, 1968; Levy and Chelune, 2007; Arnott et al., 2010; Lepow et al., 2010).

Performance in ADL is evaluated by the Barthel Index (BI). BI assesses the degree of independence in doing daily tasks in the elderly through 10 variables (bowel, bladder, grooming, toilet use, feeding, transfer, mobility, dressing, stairs, and bathing). How to score each question is as follows: 0: unable, 1: needs help, and 2: independent, and total score range is 0–20 and a higher score means the older person is more independent in doing daily tasks (Sinoff and Ore, 1997). This test was evaluated for the Iranian population by Hormozi et al. (2019).

We used a validated test of the Lawton Instrumental Activities of Daily Living (IADL) scale for Iranians(Mehraban et al., 2014) that was originally created by Lawton and Brody in 1969 to survey the complex ADLs essential for living within the community. It has got eight questions to assess competence in abilities such as using telephone, shopping, food preparation, housekeeping, laundry, mode of transportation, responsibility for own medications, and handling finance. IADL performance usually declines before ADL function such as eating, using the toilet, and bathing. IADL may recognize the early decline in physical or cognitive activity or both in elderly adults who seem vigorous and healthy (Lawton et al., 1969; Ward et al., 1998; Graf, 2008). Doing this test takes 10–15 min with the score ranging between 0 (low function) and 8 (high function). Each capacity is measured by the scale depending on either cognitive or physical function, even though all require a few degrees of both (Ng et al., 2006).

The Generalized Anxiety Disorder (GAD-7) scale contains seven items based on the Diagnostic and Statistical Manual of Mental Disorders-IV (DSM-IV) to evaluate anxiety in both the primary care setting and the general population. GAD-7 was validated by Naeinian et al. (2011) in for the Iranian population (Sapra et al., 2020). Each question has got a score range from 0 to 3 (0 = not at all, 1 = several days, 2 = more than half the days, and 3 = nearly every day) with a total score between 0 and 21, with higher scores indicating more anxiety in the elderly. Total scores of 5, 10, and 15 are considered cutoff for mild, moderate, and severe anxiety (Sapra et al., 2020).

### Statistical analyses

Based on a study by Kobayashi et al. (2019), the sample size was calculated with 95% confidence interval and 80% power using the following formula:


$$n=\frac{\left(\delta_{1}^{2}+\delta_{2}^{2}\right)\,{\left(Z_{1-\frac{\alpha }{2}}+Z_{1-\beta }\right)}^{2}}{{\left(\mu_{1}-\mu_{2}\right)}^{2}}.$$

Considering 20% probable withdrawal, 30 subjects were enrolled in each group. Data were analyzed using the Statistical Package for the Social Sciences (SPSS version 26; SPSS Inc., Chicago, IL, USA). We used a one-way ANOVA for quantitative variables and χ^2^ test for qualitative variables. A linear mixed-effects model (using an unstructured model) was used to determine the independent effects of interventions on outcomes over time (baseline and 12 weeks) with fixed effects for intervention groups, along with a random effect for participants. When the difference was significant (*P* < 0.05), *post hoc* pairwise comparison tests were performed.

## Results

### Characteristics of the patients

**FIGURE 1:**
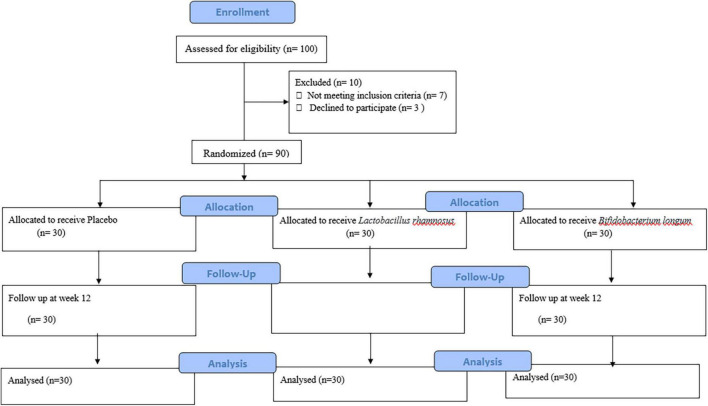
The study consort flowchart.

A total of 90 patients were included in the study and were randomly divided into three groups, namely, placebo (*n* = 30), *L. rhamnosus* (*n* = 30), and *B. longum* (*n* = 30) (Figure 1). Participants’ baseline clinical and demographic characteristics are shown in Table 1. No patients reported serious side effects while taking probiotic supplements. All the patients completed the trial and there were not any significant differences between baseline characteristics. The mean and standard deviation (SD) of MMSE, CFT, ADL, IADL, and GAD scores before and after treatment with probiotics are reported in Table 2.

**TABLE 1:** Baseline characteristics of patients in the probiotic groups and the placebo group.*

	Placebo (*n* = 30)	*Lactobacillus rhamnosus* (*n* = 30)	*Bifidobacterium longum* (*n* = 30)	*F*	*P*-value^a^
Age	67.77 ± 7.9	67.93 ± 7.8	67.90 ± 7.9	0.004	0.99
**Gender**					
Female	14 (33.3%)	14 (33.3%)	14 (33.3%)	<0.0001	1.00
Male	16 (33.3%)	16 (33.3%)	16 (33.3%)		
Education					
Illiterate	23 (31.5%)	23 (31.5%)	27 (37%)	2.32	0.31
Educated	7 (41.2%)	7 (41.2%)	3 (17.6%)		
Type of Alzheimer					
Mild Alzheimer	17 (30.9%)	15 (27.3%)	23 (41.8%)	4.86	0.08
Moderate Alzheimer	13 (37.1%)	15 (42.9%)	7 (20.0%)		
**Weight**					
Weight at baseline study (kg)	62.27 ± 6.8	67.07 ± 6.6	65.97 ± 7.1	0.32	0.73
Weight at end-of-trial (kg)	66.57 ± 6.5	68.83 ± 7.0	68.33 ± 6.0		
Weight change	−0.7 ± 2.32	1.76 ± 2.06	2.3 ± 2.15		
**BMI**					
BMI at baseline study (kg/m^2^)	23.93 ± 2.2	23.71 ± 1.4	23.79 ± 2.4	0.08	0.92
BMI at end-of-trial (kg/m^2^)	23.69 ± 2.2	24.33 ± 1.4	24.64 ± 1.8		
BMI change	−0.23 ± 0.8	0.61 ± 0.76	0.84 ± 0.81		
AChEIs	17 (30.9%)	15 (27.3%)	23(41.8%)	4.86	0.08
MMSE	15.47 ± 3.03	15.73 ± 3.89	15.50 ± 3.22	0.05	0.94
CFT	5.40 ± 2.14	5.57 ± 1.77	5.73 ± 1.63	0.24	0.78
ADL	15.80 ± 2.68	15.83 ± 3.73	15.37 ± 3.02	0.20	0.81
IADL	3.00 ± 2.00	2.40 ± 1.99	2.5 ± 1.73	4.45	0.14
GAD-7	10.00 ± 1.42	11.50 ± 2.20	11.73 ± 2.47	3.56	0.20

*Values are the mean ± SD and *P* < 0.05 is considered significant. ^a^One-way ANOVA for quantitative variables and χ^2^ test for qualitative variables. BMI, body mass index; AChEIs, acetylcholinesterase inhibitors; MMSE, the Mini-Mental State Examination; CFT, the categorical verbal fluency test; ADL, Activities of Daily Living; IADL, Lawton Instrumental Activities of Daily Living; GAD-7, the Generalized Anxiety Disorder scale.

**TABLE 2:** Mini-Mental State Examination, CFT, ADL, IADL, and GAD scores of patients in the intervention groups and the placebo group over the study period.*^a^*

Variables	Time	Groups		

		Placebo	I1*	I2^#^
		
MMSE_Orientation		Mean ± SD	Mean ± SD	Mean ± SD
	Baseline	5.87 ± 1.61	6.37 ± 2.02	6.30 ± 1.60
	12-weeks	5.17 ± 1.57	6.97 ± 1.95	7.03 ± 1.45
MMSE_Registration				
	Baseline	2.83 ± 0.37	2.87 ± 0.34	2.87 ± 0.34
	12-weeks	2.57 ± 0.56	2.90 ± 0.40	3.00 ± 0.001
MMSE_Attention				
	Baseline	0.17 ± 0.59	0.17 ± 0.37	0.07 ± 0.25
	12-weeks	0.13 ± 0.57	0.6 ± 0.77	0.43 ± 0.62
MMSE_Recall				
	Baseline	1.50 ± 0.86	1.03 ± 0.76	1.07 ± 0.74
	12-weeks	1.03 ± 0.76	2.07 ± 0.90	2.37 ± 0.76
MMSE_Language				
	Baseline	5.03 ± 1.06	5.20 ± 1.15	5.07 ± 1.14
	12-weeks	4.83 ± 1.02	5.33 ± 1.09	5.47 ± 0.93
MMSE_Visuospatial				
	Baseline	0.07 ± 0.25	0.10 ± 0.30	0.13 ± 0.34
	12-weeks	0.03 ± 0.18	0.20 ± 0.40	0.37 ± 0.49
MMSE_Total				
	Baseline	15.47 ± 3.03	15.73 ± 3.89	15.50 ± 3.22
	12-weeks	13.77 ± 3.38	18.10 ± 4.45	18.67 ± 3.05
CFT				
	Baseline	5.40 ± 2.14	5.57 ± 1.77	5.73 ± 1.63
	12-weeks	4.33 ± 2.00	7.97 ± 2.17	8.57 ± 2.12
ADL				
	Baseline	15.80 ± 2.68	15.83 ± 3.73	15.37 ± 3.02
	12-weeks	15.27 ± 2.50	16.03 ± 4.23	16.10 ± 3.33
IADL				
	Baseline	3.00 ± 2.00	2.40 ± 1.99	1.53 ± 1.73
	12-weeks	2.30 ± 1.96	3.07 ± 2.11	2.73 ± 1.6
GAD-7				
	Baseline	9.90 ± 1.42	11.50 ± 2.20	11.73 ± 2.47
	12-weeks	12 ± 2.81	6.97 ± 1.15	7.33 ± 1.18

^*a*^Values are the mean ± SD and *P* < 0.05 is considered significant. *Lactobacillus rhamnosus HA-114. ^#^*Bifidobacterium longum* R0175. MMSE, the Mini-Mental State Examination; CFT, the categorical verbal fluency test; ADL, Activities of Daily Living; IADL, Lawton Instrumental Activities of Daily Living; GAD-7, the Generalized Anxiety Disorder scale.

### Cognition

A significant effect of treatment for MMSE total score was found (*P*_Time × Group_ < 0.0001), with *post hoc* comparisons identifying significantly more improvement in the *B. longum* intervention group (differences: 4.86, 95% CI: 3.91–5.81; *P* < 0.0001) compared with the placebo and *L. rhamnosus* intervention groups (differences: 4.06, 95% CI: 3.11–5.01; *P* < 0.0001). There was no significant difference between the two intervention groups (differences: −0.8, 95% CI: −1.74 to 0.14; *P* = 0.09). We also compared all six domains of the MMSE, and analysis showed that orientation, registration, attention, recall, language, and visuospatial domains were all significantly different between the placebo and intervention groups. The CFT was significantly improved at the end of the trial (*P*_Time × Group_ < 0.0001). *Post hoc* comparisons indicated a significant increase in CFT score in the *L. rhamnosus* intervention group (differences: 3.46, 95% CI: 2.79–4.13; *P* < 0.0001) compared with the placebo and *B. longum* intervention groups (differences: 3.9, 95% CI: 3.22–4.57; *P* < 0.0001). There was no significant difference between the two intervention groups (differences: −0.43, 95% CI: −1.1 to 0.23; *P* = 0.2). More details are shown in Table 3.

**TABLE 3:** Treatment effect of probiotic supplements on MMSE, CFT, ADL, IADL, and GAD.^1^

Status	Parameter	I1 vs. placebo^#^				I2 vs. placebo^¥^				I1 vs. I2						

		Difference	95% CI		*P*	Difference	95% CI		*P*	Difference	95% CI		P	*P*_Time_	*P*_Group_	*P*_Time × Group_
													
			Lower	Upper			Lower	Upper			Lower	Upper				
Unadjusted	MMSE_Orientation	1.3	0.76	1.83	<0.0001	1.43	0.9	1.96	<0.0001	–1.33	–0.66	0.39	0.61	0.05	0.009	<0.0001
	MMSE_Registration	0.3	0.09	0.5	0.007	0.4	0.19	0.6	<0.0001	–0.1	–0.3	0.1	0.34	0.43	0.01	0.001
	MMSE_Attention	0.46	0.22	0.7	<0.0001	0.4	0.15	0.64	0.001	0.06	–0.17	0.3	0.58	<0.0001	0.2	<0.0001
	MMSE_Recall	1.5	1.1	1.8	<0.0001	1.7	1.39	2.14	<0.0001	–0.26	–0.64	0.1	0.16	<0.0001	0.05	<0.0001
	MMSE_Language	0.33	0.06	0.59	0.02	0.6	0.33	0.86	<0.0001	–0.26	–0.53	–0.0001	0.05	0.04	0.36	<0.0001
	MMSE_Visuospatial	0.13	–0.03	0.29	0.11	0.26	0.1	0.43	0.006	–0.13	–0.29	0.03	0.11	0.004	0.04	0.008
	MMSE_Total	2.3	0.54	4.03	0.011	2.46	0.71	4.22	0.006	0.16	–1.59	1.92	0.85	0.01	<0.0001	<0.0001
	CFT	3.46	2.79	4.13	<0.0001	3.9	3.22	4.57	<0.0001	–0.43	–1.1	0.23	0.2	<0.0001	<0.0001	<0.0001
	ADL	0.73	0.05	1.4	0.04	1.26	0.59	1.94	<0.0001	–0.53	–1.2	0.14	0.11	0.33	0.89	0.001
	IADL	1.36	0.77	1.95	<0.0001	1.9	1.3	2.49	<0.0001	–0.53	–1.12	0.05	0.07	0.002	0.39	<0.0001
	GAD-7	–6.63	–7.98	–5.2	<0.0001	–6.5	–7.85	–5.14	<0.0001	–0.13	–1.48	1.21	0.84	<0.0001	<0.0001	<0.0001
Adjusted*	MMSE_Orientation	1.02	0.53	1.51	<0.0001	1.07	0.57	1.57	<0.0001	–0.05	–0.53	0.41	0.81	0.44	0.02	<0.0001
	MMSE_Registration	0.25	0.05	0.46	0.022	0.34	0.13	0.55	0.003	–0.08	–0.29	0.11	0.39	0.21	0.02	0.004
	MMSE_Attention	0.43	0.19	0.68	0.003	0.36	0.11	0.6	0.006	0.07	–0.16	0.31	0.54	0.26	<0.0001	0.001
	MMSE_Recall	1.36	1.01	1.72	<0.0001	1.6	1.24	1.95	<0.0001	–0.23	–0.58	0.11	0.19	<0.0001	0.1	<0.0001
	MMSE_Language	0.17	–0.1	0.44	0.22	0.39	0.11	0.67	0.021	–0.22	–0.49	0.04	0.15	0.55	0.46	0.02
	MMSE_Visuospatial	0.12	–0.03	0.29	0.13	0.25	0.09	0.42	0.009	–0.13	–0.29	0.03	0.13	0.006	0.05	0.01
	MMSE_Total	3.28	2.47	4.08	<0.0001	3.86	3.04	4.69	<0.0001	–0.58	–1.35	0.18	0.13	0.05	0.009	<0.0001
	CFT	3.2	2.57	3.84	<0.0001	3.57	2.92	4.21	<0.0001	–0.36	–0.98	0.25	0.24	<0.0001	<0.0001	<0.0001
	ADL	0.11	–0.46	0.7	0.68	0.48	–0.11	1.09	0.28	–0.36	–0.92	0.19	0.28	0.19	0.9	0.24
	IADL	1.05	0.49	1.61	<0.0001	1.5	0.93	2.06	<0.0001	–0.44	–0.99	0.09	0.105	0.04	0.29	<0.0001
	GAD-7	–6.44	–7.77	–5.12	<0.0001	–6.26	–7.59	–4.93	<0.0001	–0.18	–1.5	1.13	0.783	<0.0001	<0.0001	<0.0001

^1^Values are the mean ± 95% CI and *P* < 0.05 is considered significant. *Adjusted based on education, type of Alzheimer’s disease, BMI, and acetylcholinesterase inhibitors (AChEIs). ^#^*Lactobacillus rhamnosus* HA-114. ^¥^*Bifidobacterium longum* R0175. MMSE, Mini-Mental State Examination; CFT, categorical verbal fluency test; ADL, Activities of Daily Living; IADL, Lawton Instrumental Activities of Daily Living; GAD-7, Generalized Anxiety Disorder scale.

### Physical activity

The effect of probiotic supplement on ADL score was not significant (*P*_Time × Group_ = 0.24). Although supplementation with *B. longum* compared with *L. rhamnosus* caused a greater increase in the ADL score, this change was not significant (differences: −0.53, 95% CI: −1.2 to 0.14; *P* = 0.11).

The IADL scale was demonstrated to improve significantly in the intervention groups compared to the placebo group (*P*_Time × Group_ < 0.0001). Compared to the placebo group, *L. rhamnosus* and *B. longum* intervention increased IADL significantly (differences: 1.05, 95% CI: 0.49–1.61; *P* < 0.0001; differences: 1.5, 95% CI: 0.93–2.06; *P* < 0.0001, respectively). There was no significant difference between the two intervention groups (differences: −0.44, 95% CI: −0.99 to 0.09; *P* = 0.10).

### Anxiety

The GAD-7 scale significantly improved after supplementation with probiotics compared with the placebo (*P*_Time × Group_ < 0.0001). Within group, comparisons showed a significant improvement in the *L. rhamnosus* intervention group (differences: −6.44, 95% CI: −7.77 to −5.12; *P* < 0.0001) compared with the placebo and *B. longum* intervention groups (differences: −6.26, 95% CI: −7.59 to −4.93; *P* < 0.0001). Although supplementation with *B. longum* compared with *L. rhamnosus* caused a greater increase in the GAD score, this change was not significant (differences: −0.18, 95% CI: −1.5 to 1.13; *P* = 0.78).

## Discussion

This present trial that investigated the effect of probiotic supplementation on cognitive status in patients with mild and moderate AD indicated a significant improvement in the MMSE total score as well as CFT, IADL, and GAD-7 in response to probiotic supplementation.

Alzheimer’s disease is a progressive neurodegenerative disease with memory decline, cognition impairment, and behavioral disorders and has become a worldwide health problem in the aging population. Although the cause of this disease is not known, it seems that two toxic misfolded proteins (amyloid-β and tau proteins) cause AD by increasing oxidative stress, neuroinflammation, and synaptic impairment and, thereby, accelerate neuronal death. Sirtuin 1 (SIRT1) is a potent therapeutic strategy against neurodegenerative diseases by downregulating Rho-associated coiled-coil-containing protein kinase 1 (ROCK) expression, and promoting anti-amyloidogenic cleavage of Aβ protein precursors by α-secretase is shown to be thereby inhibiting the aggregation of toxic proteins (Mishra et al., 2021). Since the drug’s therapeutic potential is controversial and its use is not without complications and side effects, thus, the recent paradigm shifts toward natural products and their derivatives. These natural compounds are well tolerated with minimal to no side effects. They are readily available and have excellent bioavailability and some of these compounds can affect the upregulation of SIRT1 (Ghosh et al., 2020).

At present, the gastrointestinal tract with its commensal microbiome is one of the most interesting and discussed topics in neurodegenerative diseases. However, there are few trials on the effect of probiotic supplementation, especially with single strain on the brain.

Consistent with our findings, Musa et al. (2017) showed that supplementation with *Lactobacillus fermentum* LAB9 or *Lactobacillus casei* of fermented cow’s milk had a neuroprotective effect and memory improvement through attenuation of lipopolysaccharide, inhibition of acetylcholinesterase (AChE), and antioxidative activities. Besides, animal studies have shown that supplementation with *Lactobacillus plantarum* significantly improved learning and memory abilities and stabilized the structural and functional integrity of the biological neuron membrane (Peng et al., 2014; Mallikarjuna et al., 2016; Nimgampalle and Kuna, 2017; Athari Nik Azm et al., 2018). Kobayashi et al. (2017) indicated that administration of *Bifidobacterium breve* strain A1 in AD model mice can prevent cognitive dysfunction induced by amyloid-β; antioxidant and neuroprotective effects of a probiotic formulation (namely SLAB51) have also been indicated in an SD mouse model (Bonfili et al., 2018). Leblhuber et al. (2018) showed that 4 weeks of multispecies supplementation in 20 patients with AD had a neuroprotective effect through increasing neopterin and kynurenine to tryptophan ratio as well as gut bacteria composition improvement; clock drawing test (CDT) also significantly increased. Akbari et al. (2016) indicated a significant improvement in the MMSE score by using multispecies probiotic milk (2 × 10^9^ CFU) for 12 weeks in 60 individuals with AD. Kobayashi et al. (2019) showed that supplementation with *B. breve A1* > 2.0 × 10^10^ CFU for 12 weeks in 117 older adults with subjective memory complaints produced a significant improvement in MMSE total score due to its anti-inflammatory effect through suppressing the hippocampal gene expression of inflammation-related genes. Agahi et al. (2018) revealed that multispecies probiotic supplementation (3 × 10^9^) for 12 weeks in 48 (25 patients with AD) subjects had no significant effect on Test Your Memory (TYM) score. In addition to the small number of participants and their old age, another possible reason is that 83.5% of patients had severe AD, making cognitive improvement more difficult.

There are some mechanisms explaining the effects of probiotic supplementation on cognition status. Studies have shown that a chronic neuroinflammatory state with the release of pro-inflammatory cytokines has been seen in patients with AD, which seems to correlate with constant accumulation of Aβ in neurons. It seems that this neuroinflammation occurs due to an alteration of the balance of gut microbiota (dysbiosis), which can adversely affect neuronal activity. In contrast, Alzheimer’s drugs can temporarily relieve the symptoms of the disease, but in the long-term, they adversely affect the gut microbiome, making the disease worse. Therefore, adjuvant treatment with probiotics may prevent or even cure intestinal dysbiosis, thus allowing the therapeutic effects of AD drugs to be more fully exploited (Jiang et al., 2017; Hort et al., 2020). Ingestion of probiotics in subjects with AD balances and modifies the gut microbiota to produce short-chain fatty acids (SCAFs) required for the integrity of the gut and blood–brain barrier (BBB) and other metabolites such as neurotransmitters and precursors like noradrenalin and tryptophan or γ-gamma-aminobutyric acid (GABA). These metabolites are essential for neuropsychiatric status in aging people (Leblhuber et al., 2018). Probiotics have also been reported to suppress oxidative stress and inflammation by increasing enzymes such as superoxide dismutase, glutathione peroxidase, and hs-CRP (Agahi et al., 2018). This trial indicated that the score of IADL improved significantly, whereas the score of ADL did not show any significant change. IADL performance usually declines before ADL functions such as eating, using the toilet, and bathing (Ward et al., 1998; Graf, 2008). It seems that significant changes in ADL scores may require long-term trials.

Despite broadening our understanding of the effect of probiotics on AD, this study had some limitations. For instance, we did not assess changes in the gut microbiota; therefore, it remained unclear what specific strains were modified by probiotic supplementation. Yet, the most important strengths of this study were its design as a three-arm study and the simultaneous assessment of two different single-strain probiotics.

## Conclusion

This study demonstrated that 12-week probiotic supplementation compared with placebo had favorable effects on the cognitive status, anxiety, and instrumental daily activity of patients with AD; however, this supplement had no effect on ADL. We concluded that adjuvant treatment with probiotics in these patients is beneficial and can enhance drug’s efficacy, slow the disease exacerbation process, and maintain the patient’s quality of life for an extended period, but more clinical trials are needed in this respect.

## Data availability statement

The raw data supporting the conclusions of this article will be made available by the authors, without undue reservation.

## Ethics statement

This study was done according to the Helsinki Declaration of 1975, also approved by the Ethics Committee of Tehran University of Medical Sciences and registered on the Iranian Website for Registration of Clinical Trials IRCT (IRCT number: 20210513051277N1). The patients/participants provided their written informed consent to participate in this study.

## Author contributions

KD was the guarantor. CA and ZV wrote the manuscript and collected the data. SS-B interpreted the data and provided professional comments. FE revised the manuscript. All authors contributed to the article and approved the submitted version.

## References

[B1] Agahi A. Hamidi G. A. Daneshvar R. Hamdieh M. Soheili M. Alinaghipour A. (2018). Does severity of Alzheimer’s disease contribute to its responsiveness to modifying gut microbiota? A double blind clinical trial. *Front. Neurol.* 9:662. 10.3389/fneur.2018.00662 30158897 PMC6104449

[B2] Akbari E. Asemi Z. Daneshvar Kakhaki R. Bahmani F. Kouchaki E. Tamtaji O. R. (2016). Effect of probiotic supplementation on cognitive function and metabolic status in Alzheimer’s disease: A randomized, double-blind and controlled trial. *Front. Aging Neurosci.* 8:256. 10.3389/fnagi.2016.00256 27891089 PMC5105117

[B3] Alzheimer’s Association (2010). 2010 Alzheimer’s disease facts and figures. *Alzheimers Dement.* 6 158–194. 20298981 10.1016/j.jalz.2010.01.009

[B4] Arnott W. L. Chenery H. J. Angwin A. J. Murdoch B. E. Silburn P. A. Copland D. A. (2010). Decreased semantic competitive inhibition in Parkinson’s disease: Evidence from an investigation of word search performance. *Int. J. Speech Lang. Pathol.* 12 437–445. 10.3109/17549507.2010.492875 20602578

[B5] Athari Nik Azm S. Djazayeri A. Safa M. Azami K. Ahmadvand B. Sabbaghziarani F. (2018). Lactobacilli and bifidobacteria ameliorate memory and learning deficits and oxidative stress in β-amyloid (1–42) injected rats. *Appl. Physiol. Nutr. Metab.* 43 718–726. 29462572 10.1139/apnm-2017-0648

[B6] Azm S. A. N. Djazayeri A. Safa M. Azami K. Djalali M. Sharifzadeh M. (2017). Probiotics improve insulin resistance status in an experimental model of Alzheimer’s disease. *Med. J. Islamic Republic Iran* 31:103. 10.14196/mjiri.31.103 29951404 PMC6014785

[B7] Benton A. L. (1968). Differential behavioral effects in frontal lobe disease. *Neuro Svchol.* 6:60.

[B8] Bonfili L. Cecarini V. Cuccioloni M. Angeletti M. Berardi S. Scarpona S. (2018). SLAB51 probiotic formulation activates SIRT1 pathway promoting antioxidant and neuroprotective effects in an AD mouse model. *Mol. Neurobiol.* 55 7987–8000. 10.1007/s12035-018-0973-4 29492848 PMC6132798

[B9] Clark T. M. Callam C. S. Paul N. M. Stoltzfus M. W. Turner D. (2020). Testing in the time of COVID-19: A sudden transition to unproctored online exams. *J. Chem. Educ.* 97 3413–3417.

[B10] Clemmensen F. K. Hoffmann K. Siersma V. Sobol N. Beyer N. Andersen B. B. (2020). The role of physical and cognitive function in performance of activities of daily living in patients with mild-to-moderate Alzheimer’s disease–a cross-sectional study. *BMC Geriatr.* 20:513. 10.1186/s12877-020-01926-9 33246408 PMC7693499

[B11] Diniz B. S. Yassuda M. S. Nunes P. V. Radanovic M. Forlenza O. V. (2007). Mini-mental State examination performance in mild cognitive impairment subtypes. *Int. Psychogeriatr.* 19 647–656. 17502007 10.1017/S104161020700542X

[B12] Folstein M. F. Folstein S. E. McHugh P. R. (1975). “Mini-mental state”: A practical method for grading the cognitive state of patients for the clinician. *J. Psychiatr. Res.* 12 189–198. 10.1016/0022-3956(75)90026-6 1202204

[B13] Ghosh S. Durgvanshi S. Agarwal S. Raghunath M. Sinha J. K. (2020). Current status of drug targets and emerging therapeutic strategies in the management of Alzheimer’s disease. *Curr. Neuropharmacol.* 18 883–903. 32348223 10.2174/1570159x18666200429011823 PMC7569315

[B14] Graf C. (2008). The Lawton instrumental activities of daily living scale. *AJN Am. J. Nurs.* 108 52–62. 10.1097/01.NAJ.0000314810.46029.74 18367931

[B15] Hazar N. Seddigh L. Rampisheh Z. Nojomi M. (2016). Population attributable fraction of modifiable risk factors for Alzheimer disease: A systematic review of systematic reviews. *Iran. J. Neurol.* 15:164. 27648178 PMC5027152

[B16] Henry J. D. Crawford J. R. Phillips L. H. (2004). Verbal fluency performance in dementia of the Alzheimer’s type: A meta-analysis. *Neuropsychologia* 42 1212–1222. 15178173 10.1016/j.neuropsychologia.2004.02.001

[B17] Hormozi S. Alizadeh-Khoei M. Sharifi F. Taati F. Aminalroaya R. Fadaee S. (2019). Iranian version of barthel index: Validity and reliability in outpatients’ elderly. *Int. J. Prev. Med.* 10:130. 10.4103/ijpvm.IJPVM_579_18 31516671 PMC6710921

[B18] Hort J. Valis M. Angelucci F. (2020). Administration of pre/probiotics with conventional drug treatment in Alzheimer’s disease. *Neural Regen. Res.* 15:448. 10.4103/1673-5374.266057 31571653 PMC6921357

[B19] International AsD (2019). *World Alzheimer report 2019: Attitudes to dementia.* London: Alzheimer’s Disease International.

[B20] Jack C. R. Jr. Albert M. S. Knopman D. S. McKhann G. M. Sperling R. A. Carrillo M. C. (2011). Introduction to the recommendations from the national institute on aging-Alzheimer’s association workgroups on diagnostic guidelines for Alzheimer’s disease. *Alzheimers Dement.* 7 257–262. 10.1016/j.jalz.2011.03.004 21514247 PMC3096735

[B21] Jiang C. Li G. Huang P. Liu Z. Zhao B. (2017). The gut microbiota and Alzheimer’s disease. *J. Alzheimers Dis.* 58 1–15. 28372330 10.3233/JAD-161141

[B22] Kincaid H. J. Nagpal R. Yadav H. (2021). Diet-microbiota-brain axis in Alzheimer’s disease. *Ann. Nutr.Metab.* 77 21–27. 10.1159/000515700 33906194 PMC10202336

[B23] Kobayashi Y. Kuhara T. Oki M. Xiao J.-Z. (2019). Effects of Bifidobacterium breve A1 on the cognitive function of older adults with memory complaints: A randomised, double-blind, placebo-controlled trial. *Benef. Microbes* 10 511–520. 10.3920/BM2018.0170 31090457

[B24] Kobayashi Y. Sugahara H. Shimada K. Mitsuyama E. Kuhara T. Yasuoka A. (2017). Therapeutic potential of Bifidobacterium breve strain A1 for preventing cognitive impairment in Alzheimer’s disease. *Sci. Rep.* 7 1–10. 10.1038/s41598-017-13368-2 29044140 PMC5647431

[B25] Lawton M. Brody E. Médecin U. (1969). Instrumental activities of daily living (IADL). *Gerontologist* 9 179–186. 5349366

[B26] Leblhuber F. Steiner K. Schuetz B. Fuchs D. Gostner J. M. (2018). Probiotic supplementation in patients with Alzheimer’s dementia-an explorative intervention study. *Curr. Alzheimer Res.* 15 1106–1113. 10.2174/1389200219666180813144834 30101706 PMC6340155

[B27] Lepow L. Van Sweringen J. Strutt A. M. Jawaid A. MacAdam C. Harati Y. (2010). Frontal and temporal lobe involvement on verbal fluency measures in amyotrophic lateral sclerosis. *J. Clin. Exp. Neuropsychol.* 32 913–922. 20390792 10.1080/13803391003596439

[B28] Levy J. A. Chelune G. J. (2007). Cognitive-behavioral profiles of neurodegenerative dementias: Beyond Alzheimer’s disease. *J. Geriatr. Psychiatry Neurol.* 20 227–238. 18004009 10.1177/0891988707308806

[B29] Lezak M. D. Howieson D. B. Loring D. W. Fischer J. S. (2004). *Neuropsychological assessment.* Oxford: Oxford University Press.

[B30] Mallikarjuna N. Praveen K. Yellamma K. (2016). Role of *Lactobacillus plantarum* MTCC1325 in membrane-bound transport ATPases system in Alzheimer’s disease-induced rat brain. *BioImpacts BI* 6:203. 10.15171/bi.2016.27 28265536 PMC5326668

[B31] Mckhann G. (1984). Report of the NINCDS-ADRDA work group under the auspices of department of health and human service task force on Alzheimer’s disease. *Neurology* 34 939–944. 10.1212/wnl.34.7.939 6610841

[B32] Mehraban A. H. Soltanmohamadi Y. Akbarfahimi M. Taghizadeh G. (2014). Validity and reliability of the persian version of lawton instrumental activities of daily living scale in patients with dementia. *Med. J. Islamic Republic Iran.* 28:25. 25250267 PMC4153527

[B33] Mendez M. F. (2021). The relationship between anxiety and Alzheimer’s disease. *J. Alzheimers Dis. Rep.* 5 171–177. 33981954 10.3233/ADR-210294 PMC8075566

[B34] Mishra P. Mittal A. K. Kalonia H. Madan S. Ghosh S. Sinha J. K. (2021). SIRT1 promotes neuronal fortification in neurodegenerative diseases through attenuation of pathological hallmarks and enhancement of cellular lifespan. *Curr. Neuropharmacol.* 19 1019–1037. 10.2174/1570159X18666200729111744 32727328 PMC8686317

[B35] Monsch A. U. Bondi M. W. Butters N. Salmon D. P. Katzman R. Thal L. J. (1992). Comparisons of verbal fluency tasks in the detection of dementia of the Alzheimer type. *Arch. Neurol.* 49 1253–1258. 1449404 10.1001/archneur.1992.00530360051017

[B36] Musa N. H. Mani V. Lim S. M. Vidyadaran S. Majeed A. B. A. Ramasamy K. (2017). Lactobacilli-fermented cow’s milk attenuated lipopolysaccharide-induced neuroinflammation and memory impairment in vitro and in vivo. *J. Dairy Res.* 84 488–495. 10.1017/S0022029917000620 29154736

[B37] Naeinian M. Shairi M. Sharifi M. Hadian M. (2011). *To study reliability and validity for a brief measure for assessing generalized anxiety disorder (GAD-7).*

[B38] Newcombe F. (1969). *Missile wounds of the brain: A study of psychological deficits.* Oxford: Oxford University Press.

[B39] Ng T.-P. Niti M. Chiam P.-C. Kua E.-H. (2006). Physical and cognitive domains of the instrumental activities of daily living: Validation in a multiethnic population of Asian older adults. *J. Gerontol. Ser. A Biol. Sci. Med. Sci.* 61 726–735. 10.1093/gerona/61.7.726 16870636

[B40] Nimgampalle M. Kuna Y. (2017). Anti-Alzheimer properties of probiotic, *Lactobacillus plantarum* MTCC 1325 in Alzheimer’s disease induced albino rats. *J. Clin. Diagn Res. JCDR* 11 KC01. 10.7860/JCDR/2017/26106.10428 28969160 PMC5620801

[B41] Oelschlaeger T. A. (2010). Mechanisms of probiotic actions–a review. *Int. J. Med. Microbiol.* 300 57–62. 10.1016/j.ijmm.2009.08.005 19783474

[B42] Peng X. Meng J. Chi T. Liu P. Man C. Liu S. (2014). *Lactobacillus plantarum* NDC 75017 alleviates the learning and memory ability in aging rats by reducing mitochondrial dysfunction. *Exp. Ther. Med.* 8 1841–1846. 10.3892/etm.2014.2000 25371742 PMC4218708

[B43] PorsteinssonA.IsaacsonR.KnoxS.SabbaghM.RubinoI. (2021). Diagnosis of early Alzheimer’s disease: Clinical practice in 2021. *J. Prev. Alzheimers Dis.* 8 371–386.3410179610.14283/jpad.2021.23

[B44] Sapra A. Bhandari P. Sharma S. Chanpura T. Lopp L. (2020). Using generalized anxiety disorder-2 (GAD-2) and GAD-7 in a primary care setting. *Cureus* 12:e8224. 10.7759/cureus.8224 32582485 PMC7306644

[B45] Seniów J. Litwin M. Litwin T. Leśniak M. Członkowska A. (2009). New approach to the rehabilitation of post-stroke focal cognitive syndrome: Effect of levodopa combined with speech and language therapy on functional recovery from aphasia. *J. Neurol. Sci.* 283 214–218. 10.1016/j.jns.2009.02.336 19268976

[B46] Seyedian M. Falah M. Nourouzian M. Nejat S. Delavar A. Ghasemzadeh H. (2008). *Validity of the farsi version of mini-mental state examination.* Tehran: Scientific Journal of Medical Organization of the Islamic Republic of Iran.

[B47] Shirdel S. Esmaeeli S. Alavi K. Ghaemmaghami P. Shariat S. V. (2022). Verbal fluency performance in normal adult population in Iran: Norms and effects of age, education, and gender. *Basic Clin. Neurosci. J.* 13 129–138. 10.32598/bcn.2021.363.1 PMC9790095 36589021

[B48] Shulman K. I. Herrmann N. Brodaty H. Chiu H. Lawlor B. Ritchie K. (2006). IPA survey of brief cognitive screening instruments. *Int. Psychogeriatr.* 18 281–294. 10.1017/S1041610205002693 16466586

[B49] Sinoff G. Ore L. (1997). The barthel activities of daily living index: Self-reporting versus actual performance in the old-old (≥ 75 years). *J. Am. Geriatr. Soc.* 45 832–836. 10.1111/j.1532-5415.1997.tb01510.x 9215334

[B50] Ward G. Jagger C. Harper W. (1998). A review of instrumental ADL assessments for use with elderly people. *Rev. Clin. Gerontol.* 8 65–71.

